# Sterol 14α-demethylase mutation leads to amphotericin B resistance in *Leishmania mexicana*

**DOI:** 10.1371/journal.pntd.0005649

**Published:** 2017-06-16

**Authors:** Roy Mwenechanya, Julie Kovářová, Nicholas J. Dickens, Manikhandan Mudaliar, Pawel Herzyk, Isabel M. Vincent, Stefan K. Weidt, Karl E. Burgess, Richard J. S. Burchmore, Andrew W. Pountain, Terry K. Smith, Darren J. Creek, Dong-Hyun Kim, Galina I. Lepesheva, Michael P. Barrett

**Affiliations:** 1 Department of Biomedical Sciences, School of Veterinary Medicine, University of Zambia, Lusaka, Zambia; 2 Wellcome Centre for Molecular Parasitology, University of Glasgow, 120 University Place, Glasgow, United Kingdom; 3 Glasgow Polyomics, Wolfson Wohl Cancer Research Centre, University of Glasgow, Garscube Estate, Bearsden, Glasgow, United Kingdom; 4 Biomedical Sciences Research Complex, University of St Andrews, North Haugh, St. Andrews, Fife, United Kingdom; 5 Drug Delivery, Disposition & Dynamics, Monash Institute of Pharmaceutical Sciences, Monash University, Parkville, Victoria, Australia; 6 Centre for Analytical Bioscience, School of Pharmacy, University of Nottingham, University Park, Nottingham, United Kingdom; 7 Vanderbilt University School of Medicine, Nashville, TN, United States of America; Hunter College, CUNY, UNITED STATES

## Abstract

Amphotericin B has emerged as the therapy of choice for use against the leishmaniases. Administration of the drug in its liposomal formulation as a single injection is being promoted in a campaign to bring the leishmaniases under control. Understanding the risks and mechanisms of resistance is therefore of great importance. Here we select amphotericin B-resistant *Leishmania mexicana* parasites with relative ease. Metabolomic analysis demonstrated that ergosterol, the sterol known to bind the drug, is prevalent in wild-type cells, but diminished in the resistant line, where alternative sterols become prevalent. This indicates that the resistance phenotype is related to loss of drug binding. Comparing sequences of the parasites’ genomes revealed a plethora of single nucleotide polymorphisms that distinguish wild-type and resistant cells, but only one of these was found to be homozygous and associated with a gene encoding an enzyme in the sterol biosynthetic pathway, sterol 14α-demethylase (CYP51). The mutation, N176I, is found outside of the enzyme’s active site, consistent with the fact that the resistant line continues to produce the enzyme’s product. Expression of wild-type sterol 14α-demethylase in the resistant cells caused reversion to drug sensitivity and a restoration of ergosterol synthesis, showing that the mutation is indeed responsible for resistance. The amphotericin B resistant parasites become hypersensitive to pentamidine and also agents that induce oxidative stress. This work reveals the power of combining polyomics approaches, to discover the mechanism underlying drug resistance as well as offering novel insights into the selection of resistance to amphotericin B itself.

## Author summary

Antimicrobial resistance threatens to reverse many of the great strides made against pathogens responsible for disease. Understanding the molecular processes underlying resistance is crucial to quantifying and tackling the problem. Here we select resistance in *Leishmania* parasites to amphotericin B, an antileishmanial drug of increasing importance. We then combine genome sequencing with untargeted and targeted metabolomics analyses to identify a gene, sterol 14α-demethylase, mutation of which drives a change in sterol metabolism and loss of ergosterol, the molecular target of amphotericin B. Accumulation of a downstream intermediate of ergosterol biosynthesis indicated the enzyme itself retains activity, but the pathway to ergosterol is truncated. Expression of wild-type sterol 14α-demethylase in the resistant cells restored amphotericin B sensitivity and normal ergosterol production.

## Introduction

The leishmaniases are a complex of diseases caused by parasitic protozoa of the genus *Leishmania* which are transmitted between people via the bite of an infected sandfly [1]. The specific disease caused by the parasites depends upon which *Leishmania* species is responsible and ranges from a self-limiting cutaneous form, through a mucocutaneous disease and a frequently fatal visceral form [2]. Control is largely dependent upon the use of chemotherapy. In recent years the polyene amphotericin B (AmB) has emerged as the treatment of choice where available, particularly in the liposomal formulation which abrogates some of the toxic effects associated with the parent compound itself [3]. The specificity of AmB relates to its mode of action being mediated through a binding to the membrane sterol ergosterol, which is the primary sterol of fungal and *Leishmania* membranes, while binding with less avidity to cholesterol [3], the principal sterol of mammalian host membranes. It was suggested that AmB molecules polymerise at membranes where they bind, forming pores that cause membrane leakage to various ions and this has been proposed as a key cause of death [4], although binding to ergosterol alone is sufficient to cause death in fungi [5, 6].

AmB in a liposomal formulation, AmBisome, has emerged as a treatment of choice because of the enhanced efficacy of the drug against macrophage-resident *Leishmania* parasites and the accompanying reduction in host toxicity [7, 8]. Several trials using the drug as a combination with other leishmanicides [9] indicate that AmB containing combinations offer promise for future therapies [10]. Other trials [11] have indicated that a single injection of AmBisome is efficacious, particularly in India where other drugs, including pentavalent antimony [12] and miltefosine [13] are suffering from treatment failure and increasing resistance. The fact that the incidence of resistance to AmB in fungi has been slow to emerge, in spite of over 50 years of use [14], has underpinned a belief that the fitness costs associated with any resistance might protect against the problem [15]. However, there are increasing reports of AmB resistance in fungi [16–18]. Moreover, several reports of AmB treatment failure have been reported in leishmaniasis patients in India [19, 20] and in immunocompromised patients in France [21] and resistance to the drug has been reported to occur in at least one field isolate already [19]. Resistance in that case was proposed to relate to several phenotypic changes to the parasite, notably a change in sterol metabolism [19] and increase in defence against oxidative stress [22]. In common with several reports in *L*. *mexicana* [23] and *L*. *donovani* [19, 24], selection of resistance was associated with the replacement of ergostane-type sterols with cholestane-type sterols, the latter being less avid binders of AmB [3].

Other studies into changes occurring in selected AmB resistance in *Leishmania* point to alterations in enzymes of cellular thiol [24, 25] and ascorbate [22] metabolism leading to an enhanced resistance to oxidative stress being associated with selection. Although changes to C24-Δ-sterol methyl transferase gene expression suggested a possible genetic marker for resistance [19, 26] direct corroboration is lacking, and no specific gene mutations have yet been described that correlate unequivocally with resistance. Understanding molecular mechanisms of drug resistance provides potential biomarkers to assess the spread of resistance and can also offer routes to slow the emergence of resistance or even bypass the problem. Due to the lack of economic incentivisation for new drug development, it is essential to retain existing drugs for neglected tropical diseases, such as the leishmaniases, if we are to achieve aims of bringing the disease under control.

Here we use the complementary high throughput data approaches of metabolomics and whole genome sequencing to reveal a gene whose mutation causes resistance to AmB in *Leishmania mexicana*.

## Materials and methods

### Cell culture and AmB resistance selection

Promastigotes of *L*. *mexicana* strain MNYC/BZ/62/M379 were cultured in Homem (GIBCO) medium [27] supplemented with 10% foetal bovine serum—Gold (FBS) (PAA Laboratories GmbH) starting at a density of 1 x 10^5^ cells/ml and maintained at 27°C, passaging once every 72 hours. The cells were selected for AmB resistance by increasing concentrations of the drug, initially exposing cells to 0.0135 μM of AmB (Sigma-Aldrich) with stepwise doubling of the drug concentration to a final concentration of 0.27 μM. Cells able to grow in the presence of the drug were cloned under drug pressure by limiting dilution to 1 cell/ml in 20 ml of growth medium and plated out into 96-well plates.

### Drug sensitivity assays

Susceptibility of the cells to various drugs was determined using an adaptation of the Alamar Blue assay [28]. A starting density of 1 × 10^6^ cells/ml were incubated at 27°C in the presence of various drug concentrations for 72 hours in a 96-well microtiter plate. Resazurin (Sigma-Aldrich) in 1× phosphate-buffered saline (PBS) (Sigma-Aldrich) pH 7.4 solution was added to a concentration of 44.6 μM and cells incubated for a further 48 hours. The fluorescence of the reacted dye was measured on a FLUOstar OPTIMA (BMG LabTech, Germany) spectrometer set at excitation and emission wavelengths of 530 nm and 590 nm, respectively. The drugs used in the susceptibility assays, unless stated otherwise, were bought from Sigma-Aldrich. To assess the sensitivity to H_2_O_2_, wild-type and derived AmB resistant cells at a starting density of 2 × 10^6^ cells/ml were exposed to 20 μM, 200 μM, 500 μM and 1 mM of H_2_O_2_ [29] in growth medium in 6-well plates. The response by the two cell lines to H_2_O_2_ were compared by observation under a light microscope at different time points over 72 hours. An alternative test for H_2_O_2_ sensitivity involved glucose oxidase as described previously [30]. Briefly, 180 μl of cells at 5 x 10^5^cells/ml were plated into a 96-well plate and 20 μl of glucose oxidase solution (Sigma-Aldrich) were added in varying concentrations then tested using the AlamarBlue assay described above.

### Cell body length determination

The cell body length of *L*. *mexicana* promastigotes was determined using the SoftWoRx 5.5 software on a DeltaVision Applied Precision Olympus IX71 microscope. Smears of cells from late log phase culture were spread onto a microscope slide. The cells were fixed in absolute methanol overnight at -20°C then rehydrated with 1 ml of 1 × PBS for 10 minutes. 50 μl of PBS containing 1 μg/ml 4, 6-diamidino-2-phenylindole (DAPI) (Sigma-Aldrich) and 1% 1, 4-Diazobicyclo-(2, 2, 2) octane (DABCO) (Sigma-Aldrich) were added to stain the cells. The cell length was measured from the anterior end to the posterior end of the cell body.

### Metabolite extraction and analysis by LC-MS untargeted metabolomics

Cells were grown to mid-log phase and 1 × 10^8^ cells for each sample collected and metabolism was quenched rapidly by cooling them to 4°C in a dry ice/ethanol bath while mixing vigorously to avoid freezing and possible cell lysis [31]. Cells were separated from medium by centrifugation at 1,250g for 10 minutes at 4°C and 5 μl of supernatant was used for spent medium analysis. Metabolites were extracted from the cell pellet by addition of 200 μl of chloroform-methanol-water (1:3:1) solution and mixed vigorously at 4°C for 1 hour. The metabolites were separated from the cell debris by centrifugation at 13,000g for 5 minutes at 4°C and the samples were stored under argon gas at -80°C until analysis. Separation and mass detection of the metabolites was performed according to [32], using the DionexUltiMate3000 Liquid chromatography system using a SeQuant ZIC-HILIC column coupled to the Orbitrap Exactive mass spectrometer at Glasgow Polyomics, University of Glasgow. Raw data was processed and analyzed using the mzMatch [33] and IDEOM [34] software platforms. Metabolite identifications were given at Level 2 according to the Metabolomics Standards initiative (MSI) where accurate masses and predicted retention times were used to yield putative annotations but when retention times of authentic standards were available, the identification should be considered as Level 1 [35]. Metadata to support the identification of each metabolite is available in the IDEOM file for each study (S1 Table). It is important to note that many of the metabolite names given in the IDEOM file are generated automatically as the software provides a best match to database entries of the given mass and formula. In the absence of additional information these must be considered as putatively annotated hits; the confidence score in the column adjacent to that hit serves as a guide to this. Clearly it is beyond the scope of any study to provide authenticated annotations to many hundreds of detected compounds, but the full datasets are included in the spirit of open access data.

### Analysis of sterols by GC-MS

Mid log phase cells were taken and washed in PBS before 1.5 ml 25% KOH in 60% ethanol was added to each 100 mg cells in glass tubes. Samples were incubated at 85°C for one hour then an equal volume of *n*-heptane was added. Samples were vortexed then incubated at room temperature for 10 min. The top layer containing the sterols was transferred to a new glass vial for analysis.

One microlitre of heptane extract sample was injected into a Split/Splitless (SSL) injector at 270°C using splitless injection (1 minute) into Trace 1310 gas chromatograph (Thermo Scientific). Helium carrier gas at a flow rate of 1.2 ml/min was used for separation on a TraceGOLD TG-5SILMS 30 m length with 5 m safeguard × 0.25 mm inner diameter × 0.25 μm film thickness column (Thermo Scientific). The initial oven temperature was held at 50°C for 2 min. Separation of sterols was performed using a gradient of 20°C/min from 50 to 325°C with an 8.5 minutes final temperature hold at 325°C. Eluting peaks were transferred through an auxiliary transfer temperature of 275°C into a Q-Exactive GC mass spectrometer (Thermo Scientific). Electron ionisation (EI) was at 70 eV energy, with an emission current of 50 μA and an ion source of 230°C. A filament delay of 3.5 minutes was used to prevent excess reagents from being ionised. Full scan accurate mass EI spectrum at 60,000 resolution were acquired for the mass range 50 to 750 m/z.

Peak detection used the Xcalibur software (Thermo Scientific). Masses were compared to those in the NIST/EPA/NIH Mass Spectral Library (EI).

### Genomic DNA extraction and sequencing

Cells for genomic DNA extraction were grown to mid-log phase in a 10 ml culture and harvested by centrifugation at 1, 250g for 10 minutes and washed once in 1 × PBS. The cells were re-suspended in 500 μl NTE buffer (10 mM Tris-HCl pH 8.0; 100 mM NaCl; 5 mM EDTA) to which 25 μl of 10% SDS and 50 μl of 10 mg/ml RNase A (Sigma-Aldrich) were added and warmed to 37°C. The solution was mixed by inverting and incubated at 37°C for 30 minutes. After addition of 25 μl of 20 mg/ml pronase (Sigma-Aldrich) the lysates were incubated at 37°C overnight. The samples were then extracted twice with phenol:chloroform:isoamyl alcohol (25:24:1) (Sigma-Aldrich) and chloroform, with mixing for 5 minutes between extraction steps. The aqueous phase was obtained after centrifugation at 16,000g for 10 minutes and the DNA was precipitated with absolute ethanol and washed once with 70% ethanol. The DNA was dried in the fume hood and after being dissolved in water the concentration was determined using a NANODROP 1000 spectrophotometer (Thermo Scientific). Paired-end samples of the genomic DNA for the progenitor wild-type and derived AmB resistant cells were sequenced using Illumina GAIIx next generation DNA sequencing platform and analysed at Glasgow Polyomics, University of Glasgow. All DNA sequence information is deposited at the European Nucleotide Archive (ENA) under project number PRJEB10872.

### Expression of WT CYP51 in AmB resistant cells and GFP-tagging

The expression vector pNUS-HnN for *Crithidia fasciculata* and *Leishmania* [36] was used to express the WT sterol 14α-demethylase gene (LmxM.11.1100) fused to the His-tag at the N-terminus in AmB resistant *L*. *mexicana*. The vector pNUS-GFPcN was used for expression of both WT and N176I CYP51 with the Green Fluorescent Protein (GFP) tag at the C-terminus in both resistant and WT *L*. *mexicana*. The genes were amplified by PCR using Phusion High-Fidelity DNA polymerase (New England Biolabs). Primers for pNUS-HnN incorporating *Nde*I and *Xho*I restriction sites (underlined) for WT CYP51 were forward 5' GCATATGATGATCGGCGAGCTTCTCC3' and reverse 5'CTCGAGCTAAGCCGCCGCCTTCT3'. For expression of the WT and N176I CYP51 in pNUS-GFPcN, the forward and reverse primers were 5’CATATGATGATCGGCGAGCTTCTCCT3’ and 5’AGATCTAGCCGCCGCCTTCTTC3’, respectively, with *Nde*I and *Bgl*II restriction sites. Sterol C14-reductase (LmxM.31.2320) was expressed in pNUS-GFPcN using forward 5’CATATGATGGCAAAACGCAGAGGTACTG3’ and reverse 5’AGATCTGTATATGTACGGGAACAGCC3’ primers, respectively. The genes were initially sub-cloned into pGEM-T Easy vector (Promega) and multiplied in XL1 Blue *E*. *coli* competent cells (Promega) prior to cloning into the pNUS vectors. The presence of the gene fragments was confirmed by their PCR amplification using vector-specific primers designed from the vector sequences on http://www.ibgc.u-bordeaux2.fr/pNUS/index.html. Thus, presence of WT CYP51 in the pNUS-HnN was verified with forward 5'CATCATCATCATCACAGCAGC3' and reverse 5'GTCGAAGGAGCTCTTAAAACG3' primers, while the presence of both the WT and N176I CYP51 in pNUS-GFPcN was verified with the forward 5’TATCTTCCACTTGTCAAGCGAAT3’ and reverse 5’CCCATTCACATCGCCATCCAGTTC3’ primers. Similarly, the presence of these genes was confirmed by PCR in DNA extracted from the transfectants. The presence of mutated chromosomal CYP51 in AmB resistant *L*. *mexicana* expressing the WT CYP51 gene was confirmed by PCR amplification using a forward primer (5'CGCGAAATAGATATAAAGCACACG3') starting from 43 bp upstream of the start codon of CYP51 or 569 bp from the point mutation and a reverse primer (5'TCGCGAGCGATGATAATCTCG3') starting 213 bp downstream the mutated base resulting in a 788 bp PCR fragment. PCR amplification fragments were sequenced at Eurofins MWG Operon, Germany and aligned using CLC workbench Genomics software. All primers were purchased from Eurofins MWG Operon, Germany.

### Transfection of *L*. *mexicana* and selection for the re-expressors

*L*. *mexicana* promastigotes were grown to log phase and 1 × 10^7^ cells were harvested and washed then re-suspended in 100 μl transfection buffer (90 mM NaPO_4_ pH7.3; 5 mM KCl; 50 mM HEPES pH7.3; 0.15 mM CaCl_2_) and added to 10 μg of plasmid DNA in a cuvette before transfection with an Amaxa biosystems NucleofectorII (Lonza) using program U-033. The cells were incubated on ice for 10 minutes and then transferred into pre-warmed 10 ml of Homem supplemented with 10% FBS-Gold and left to recover for 18 hours at 27°C. G418 disulfate salt (Sigma-Aldrich) at 50 μg/ml was added to select cells carrying the plasmids. Clones of the selected cells were obtained in the presence of 50 μg/ml G418 in growth medium by limiting dilution.

### Subcellular localisation

Immunofluorescence microscopy was performed with WT and AmB resistant cell lines expressing GFP-CYP51 or tagged sterol reductase (GFP-SR) from episomal vectors. 200 μl of mid-log phase cells were collected and washed two times with PBS, fixed in 1% formaldehyde (methanol-free, Thermo Scientific) for 30 minutes. Triton X-100 (Sigma-Aldrich) was added up to final concentration 0.1% and incubated for 10 minutes, afterwards, glycine was added to the final concentration of 0.1 M and incubated for an additional 10 minutes. Cells were centrifuged, resuspended in PBS, spread on microscopy slides and left to dry. Slides were washed with PBS and blocked with PBS, 0.1% Triton X-100, 0.1% BSA (Sigma-Aldrich) for 10 minutes. Primary antibody against the ER specific chaperone BiP [37], a gift from Professor J. Bangs (University of Buffalo, New York), was applied in dilution of 1:5000, overnight, at 4°C. Subsequently, slides were washed three times with PBS and incubated with secondary anti-rabbit Alexa Fluor antibody (Molecular Probes). Following 1 hour incubation, slides were washed three times with PBS, dried and mounted with 5 μM 4’,6-diamidino-2-phenylindole (DAPI). Microscopy was performed using Axioscope, Volocity software and processed with ImageJ software. For mitochondrial staining, cells were incubated with 100 nM MitoTracker red (Molecular Probes) at 25°C for 30 minutes. Subsequently, cells were fixed as described above and mounted with DAPI.

## Results

### Selection for AmB resistant *L*. *mexicana* and its characterisation

**Fig 1:**
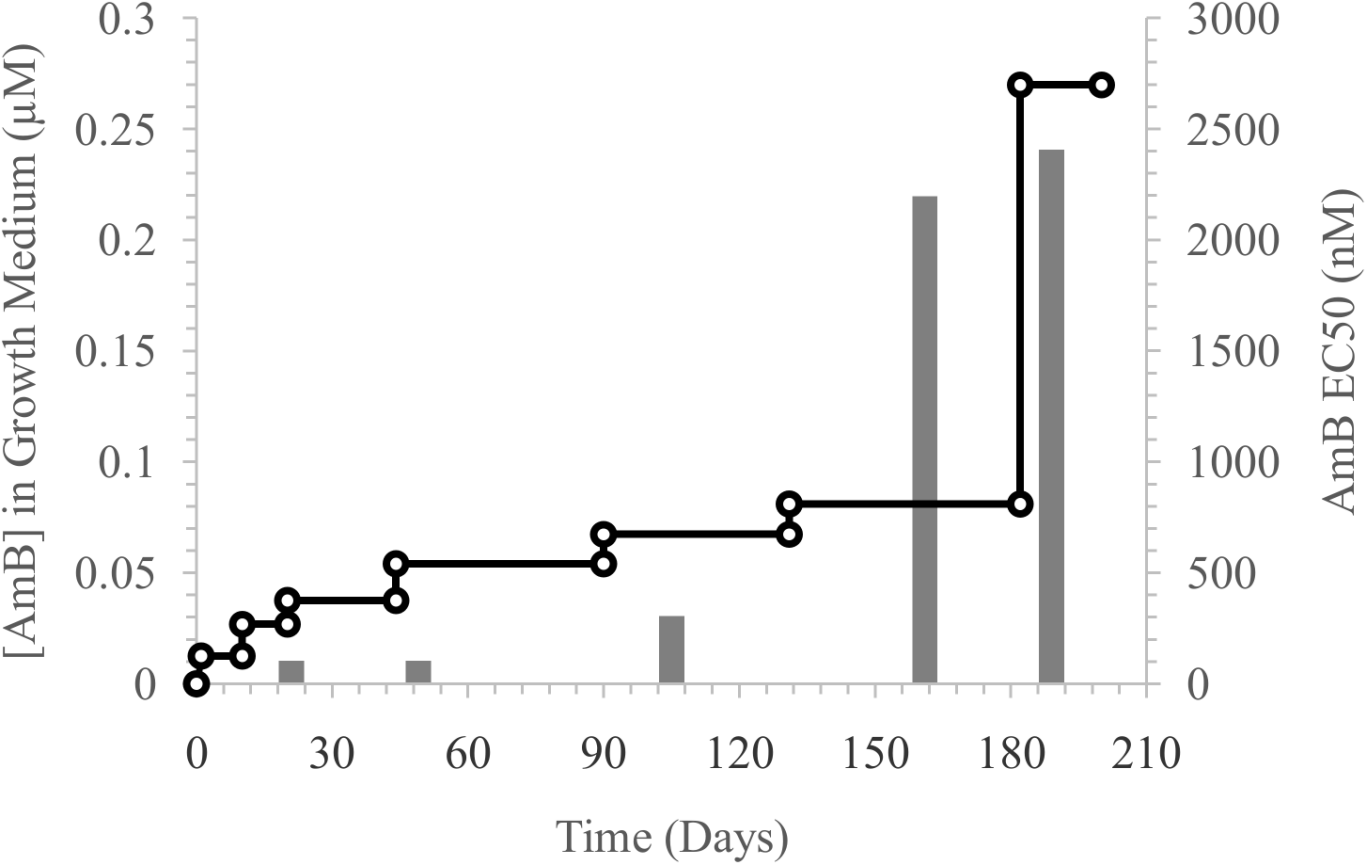
Selection of AmB resistance in *L*. *mexicana* promastigotes. AmB resistance was selected by step-wise increase in AmB concentrations in the growth medium. The open circles and left *y* axis indicate the AmB concentration in the growth medium during the selection over 182 days as shown on *x* axis. The grey bars and the right *y* axis indicate the specific EC_50_ values (representative single values of three are plotted) for AmB attained during the selection process at different times as indicated by the *x* axis.

AmB resistant *L*. *mexicana* promastigotes were selected by stepwise increase in drug concentration in culture medium over 18 passages stretching over six months. During this period, a 23-fold increase (P = 0.0007) in EC_50_ value above that of the wild-type (WT) was observed (Fig 1 and S1 Fig). Sustained growth in a drug concentration above 0.27 μM could not be achieved. The acquired AmB resistance was stable over at least 15 passages in drug free medium.

There was no appreciable difference in the growth phenotype between the resistant and the WT cells, although during the process of resistance induction, the derived AmB resistant cells required at least five passages of adaptation to a given drug concentration before they would grow at similar rates to WT.

The cells showing the highest resistance level had a significantly reduced cell body length compared to the WT cells (P < 0.0001). The late log-phase WT cells and the resistant clone had average cell body lengths of 11.16 ± 0.19 μm (n = 126) and 9.86 ± 0.16 μm (n = 126), respectively.

### Response of AmB resistant cells to other antileishmanial drugs and oxidative stress

The AmB resistant cells exhibited mild cross-resistance to potassium antimony tartrate (PAT) and miltefosine with fold change in EC_50_ values of 2.9 and 3.9 representing significant differences (P = 0.0005 and P < 0.0001, respectively) to the WT (Table 1 and S2 Fig). A marginal 1.7-fold increase in the EC_50_ value (P = 0.0007) to ketoconazole (an inhibitor of sterol synthesis at the sterol 14α-demethylase step) was also observed in the AmB resistant line.

**Table 1:** Drug sensitivity of AmB resistance cell lines in this and previous studies. While *L*. *mexicana* was used here, the quoted values are for *L*. *donovani* from Mbongo et al. (1998), and Garcia-Hernandez et al. (2012). Fold change is calculated as the ratio of EC_50_ value of the resistant cell line to WT for a given drug. EC_50_ values are expressed as means of three replicates ± SEM.

Drug	WT	AmB-R	Fold change	WT	AmB-R	Fold change	WT	AmB-R	Fold change
	EC_50_ [μM]			EC_50_ [μM]			EC_50_ [μM]		
AmB	0.10 ± 0.004	2.41 ± 0.11	23	0.70 ± 0.01	0.14 ± 0.04	2	0.10 ± 0.01	1.89 ± 0.12	18.9
Pentamidine	4.19 ± 0.27	0.3164 ± 0.008	0.08	-	-	-	2.7 ± 0.8	1.4 ± 0.2	0.52
Ketoconazole	15.06 ± 0.66	26.05 ± 0.942	1.73	-	-	-	> 1.9	0.13 ± 0.01	< 0.07
Miltefosine	5.82 ± 0.11	22.58 ± 0.20	3.88	5.84 ± 0.43	5.28 ± 0.58	0.90	-	-	-
Sb^III^ (PAT)	197.6 ± 11.79	564.6 ± 32.65	2.86	87.33 ± 5.72	74.38 ± 4.98	0.85	-	-	-
**Source**	This study			[38]			[24]		

Interestingly, the AmB resistant cells were found to be more susceptible to pentamidine (P = 0.0001) with decreases in EC_50_ values of 13.3-fold.

We also tested the effect of various reagents causing oxidative stress. Exposure of both cell lines to methylene blue, a stress inducing agent [39], showed that AmB resistant cells were far more susceptible to this agent with EC_50_ values of 0.117 ± 0.001 μM against 4.20 ± 0.22 μM for WT (P < 0.0001).

Addition of 500 μM H_2_O_2_ directly to cells induced swelling resulting in their assuming rounded shapes, and sluggish to no movement. Resistant cells recovered from exposure more slowly than WT cells (as judged by inspection of flagellar motility) and by 72 hours following exposure reached average densities of 4 × 10^6^ cell/ml whilst WT cells were at 9 × 10^6^ cells/ml.

Because H_2_O_2_ is labile, we also tested the effect of glucose oxidase in medium. Glucose oxidase produces H_2_O_2_ continuously [30] and the concentration of enzyme added to medium can, therefore, act as a surrogate for quantitation of susceptibility to the peroxide. 4.5 mU/ml of glucose oxidase were required to inhibit growth of WT cells by 50% whilst the EC_50_ for the resistant cell line was just 1.8 mU/ml, confirming the increased sensitivity to stress of the resistant cell line.

### Untargeted metabolomics reveals significant changes in sterol metabolism between WT and AmB resistant *L*. *mexicana*

**Fig 2:**
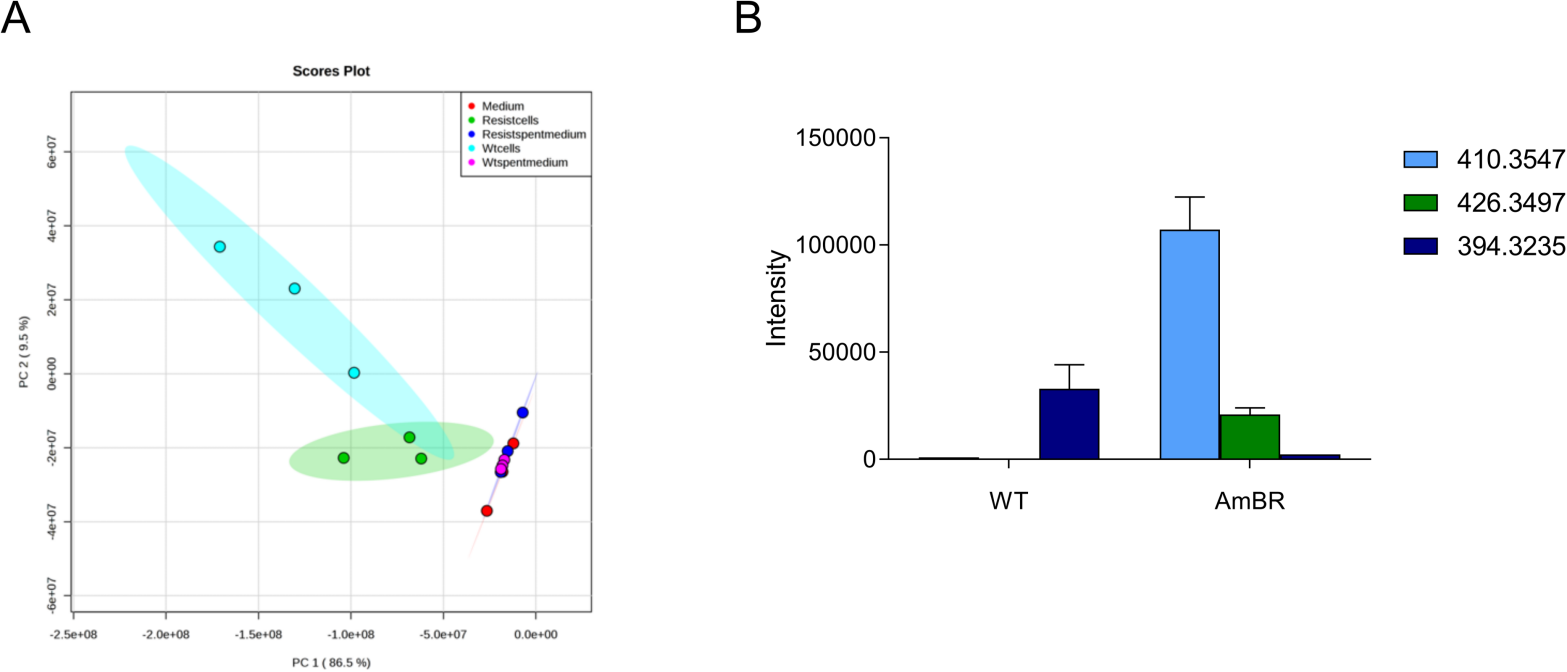
LC-MS metabolomics analysis of WT and AmB resistant *L*. *mexicana* promastigotes. A. PCA plot of the LC-MS metabolomic analysis. Each circle represents a single replicate and shaded areas indicate respective 95% confidential intervals; light blue, WT cells; green, AmB resistant cells; red, fresh medium; magenta, WT spent medium; dark blue, AmB resistant cells spent medium. B. Representative sterols as detected by LC-MS metabolomic analysis significantly changing between WT and AmB resistant cells. Mass of these metabolites is listed since specific identification is not possible by LC-MS, but formulae of 410.3547 = C_29_H_46_O; 426.3497 = C_29_H_46_O_2_; 394.3235 = C_28_H_42_0. Mean values of three replicates are plotted, error bars represent standard deviations.

Using an untargeted liquid chromatography-mass spectrometry (LC-MS) metabolomics approach we compared the two cell lines. Principal components analysis (PCA) revealed the WT and resistant lines to have clear differences (Fig 2A). Among the most significant changes were alterations around the sterol metabolic pathway. Previous studies in both *L*. *donovani* [19, 24] and *L*. *mexicana* [23] have also identified changes to sterols (specifically an increase in cholesta-5,7,24-trien-3β-ol in *L*. *donovani* and 4,14-dimethyl-cholesta-8,24-dienol and other methyl sterols in *L*. *mexicana*). A metabolite of m/z 394.32 putatively identified as ergosta-5,7,22,24(28)-tetraen-3β-ol was diminished in resistant cells compared to WT cells (Fig 2B and S1 Table) while a metabolite of m/z 410.35 putatively identified as 4,4-dimethylcholesta-8,14,24-trien-3β-ol and another with m/z 426.35 consistent with 4α-formyl-4β-methylcholesta-8-24-dien-3β-ol were more abundant in resistant cells compared to WT cells (Fig 2B and S1 Table).

While the LC-MS approach taken allows comprehensive coverage of the metabolome and is thus ideally suited to initial identification of those areas of metabolism changing in response to biological perturbation, the hydrophilic interaction liquid chromatography (HILIC)-based liquid chromatography platform is not suitable for separation and robust identification of individual lipids. Having identified that changes in sterol metabolism were key, we adopted a gas chromatography (GC)-MS approach since this methodology had been previously applied to the identification of sterol metabolism in *L*. *mexicana* [40].

**Fig 3:**
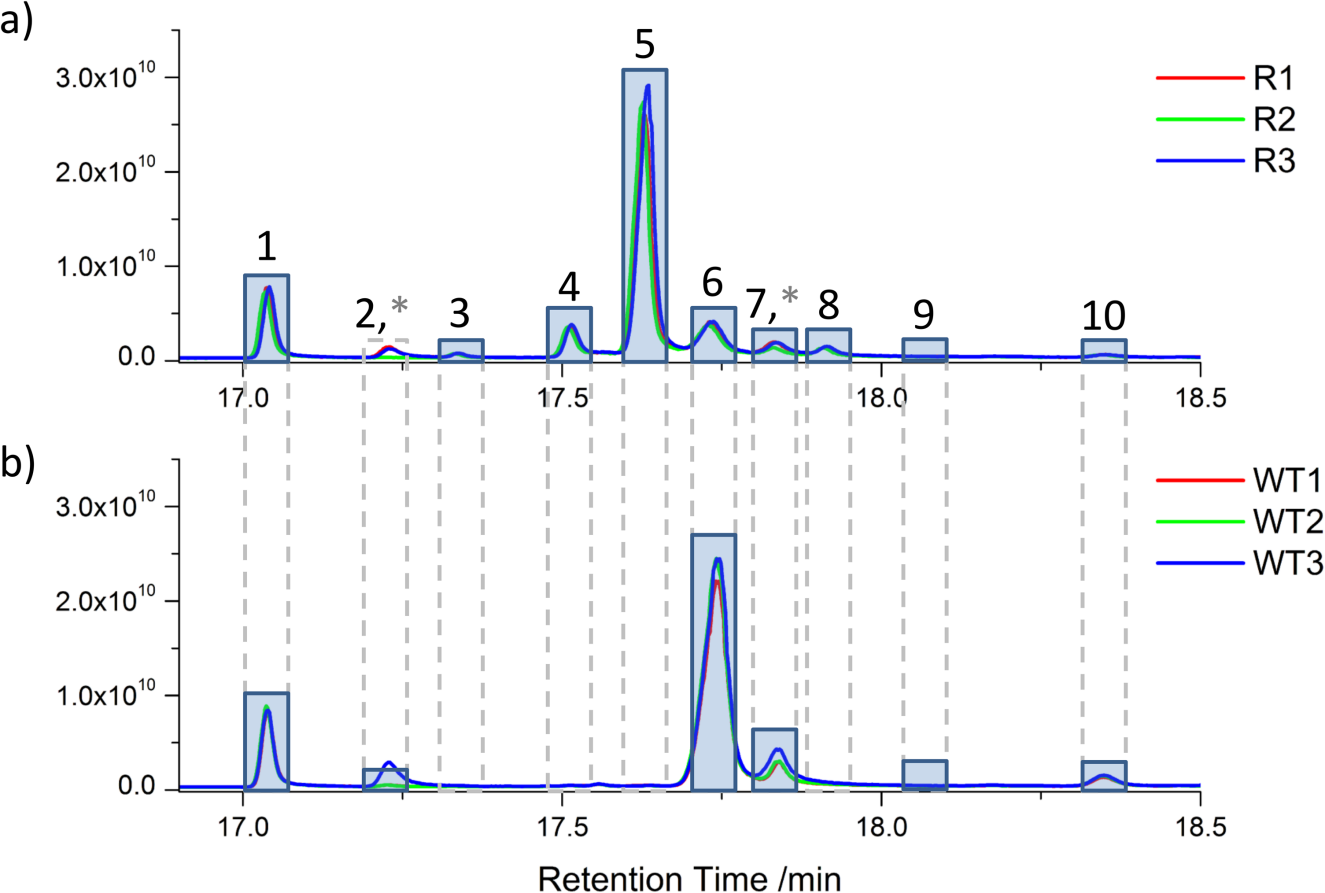
**Total ion chromatograms for a) AmB resistant (R) and b) wild type (WT).** Extracted sterols were analysed by high resolution accurate mass Q-Exactive GC Orbitrap. Nine unique sterols were identified in the retention time region from 17 to 18.4 min. The identification of these sterols is listed in Table 2. Asterisks denote polysiloxane contaminant peaks that co-elute with sterol peaks. Three replicates of each extraction show high reproducibility with regard to peak height.

Fig 3 shows chromatograms obtained from the GC-MS, and identities of the detected peaks are indicated in Table 2 based on matches with the NIST library (https://www.nist.gov/srd/nist-standard-reference-database-1a-v14). The major difference between WT and AmB resistant cells is depletion of peak 6 representing ergosterol (the most abundant sterol in WT) and concomitant increase in peak 5, corresponding with 14-methylergosta-8,24(28)-dien-3β-ol in the resistant cell line. Peak 4, not detected in WT, is abundant in resistant cells, and corresponds to 4,4-dimethylcholesta-8,14,24-trien-3β-ol, a product of the sterol 14α-demethylase reaction (note, however, that isomers of this compound exist that we cannot distinguish). In addition, peaks 3 and 8 are increased 50-fold and 80-fold, respectively, in the AmB resistant cell line, putatively identified as ergosta-5,24(28)-dien-3β-ol and 4,14-dimethylergosta-8,24(28)-dien-3β-ol. The level of cholesterol was unchanged because it is acquired from the medium rather than synthesised [41].

**Table 2:** Identification of the sterol peaks indicated in Fig 3. Identification is based on matches with the NIST library after fragmentation and the scores are indicated. Abundance relative to the total sterol content is indicated in each cell line, and the fold change of that.

Label	Base Fragment Ion (*m/z*)	Molecular Ion (*m/z*)	Fragment Formula	NIST Library Match	NIST SCORE	WT % of Total	Resistant % of Total	Fold Change
1	301.28903	386.35440	C_21_H_33_O	Cholesterol	765	0.10	0.11	1.1
2	369.31524	384.3384	C_26_H_41_O	5α-Cholesta-8,24-dien-3β-ol = zymosterol	656	0.24	0.02	0.083
3	383.33083	398.35436	C_27_H_43_O	Ergosta-5,24(28)-dien-3β-ol	616	0.02	1.02	51
4	377.32047	410.35460	C_28_H_41_			0.00	6.72	-
5	397.34652	412.37016	C_28_H_45_O	14α-Methyl-5α-ergosta-8,24(28)-dien-3β-ol	708	0.12	72.46	604
6	363.30477	396.33876	C_27_H_39_	Ergosterol	713	86.32	12.83	0.15
7	271.20583	398.35586	C_19_H_27_O			9.80	4.02	0.41
8	411.36225	426.38595	C_29_H_47_O	4α,14α-Dimethyl-5α-ergosta-8,24(28)-dien-3β-ol	694	0.02	1.63	81.5
9	411.36240	426.38596	C_29_H_47_O	Lanosterol	599	0.09	0.15	1.67
10	377.32030	410.35440	C_28_H_41_	(3β)-Stigmasta-5,7,22-trien-3-ol	727	3.28	1.05	0.32

**Fig 4:**
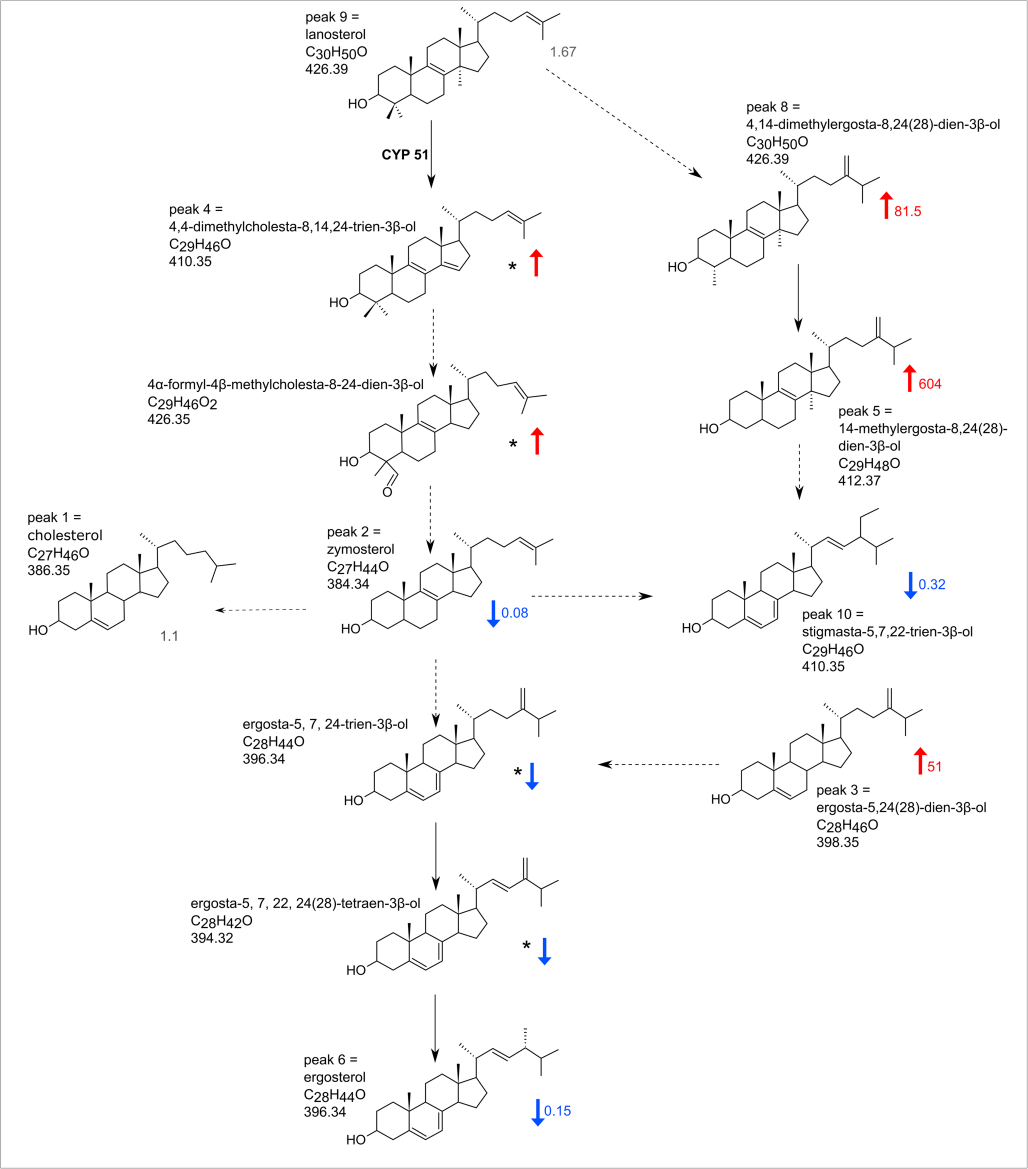
Ergosterol biosynthetic pathway with indicated sterols detected in metabolomic analyses. The pathway is based on the metacyc.org database and Roberts et al. [42], however, the exact topology of the pathway in *Leishmania* is unknown. Colour arrows and numbers indicate sterols significantly changed in AmB resistant cells when compared to WT, and their relative abundance. Asterisks mark metabolites detected by LC-MS only, thus their identification is only putative.

The detected sterols were mapped onto a pathway based on work by Roberts et al., [42] and metacyc.org database (Fig 4). Overall, components from the upper part of the pathway are accumulated whereas intermediates of the downstream steps are decreased.

Replacement of ergostane-type sterols in WT cells with cholestane-type sterols in resistant derivatives, are similar to those noted in *L*. *donovani* promastigotes selected for resistance [24] and also a field isolate of *L*. *donovani* from a refractory patient [19]. In a separate study, *L*. *mexicana* selected for resistance had also lost ergosterol, but in this case the sterol species that accumulated was 4,14-dimethylcholesta-8,24-dienol [23], indicating that there may be several distinct ways whereby loss of ergosterol synthesis can be achieved, with the accompanying accumulation of other sterols. In principle, the loss of any enzyme in the ergosterol synthetic pathway could lead to loss of production of that sterol and acquisition of resistance to AmB. For example, Pourshafie et al. [26] point to possible mutations in C-24-Δ-sterol-methyltransferase causing the increase in cholesta-5,7,24-trien-3β-ol they identified.

The related parasite *Trypanosoma brucei* accumulates exogenous cholesterol for membrane biogenesis [43] and *L*. *donovani* deficient in a cytochrome P450 enzyme related to sterol 14α-demethylase, termed CYP5122A1, produce less ergosterol than WT cells and grow less well but recover WT growth rates if medium is supplemented with exogenous ergosterol [44]. We therefore tested whether addition of exogenous ergosterol would accumulate in membranes of our resistant cells and re-sensitise them to AmB. However, addition of exogenous ergosterol (7.6 μM, as used in reference [44]) for 5 passages prior to testing drug efficacy, failed to re-sensitise, rather it further reduced sensitivity (2.84-fold increase in EC_50_ value for AmB (P = 0.0004)) which could be attributed to the drug binding ergosterol in medium.

### Genomic DNA sequencing and comparison of WT and AmB resistant *L*. *mexicana* reveals a single nucleotide polymorphism in sterol 14α-demethylase

Whole genome sequencing of the resistant line and its WT progenitor (passaged in parallel during the course of resistance selection) resulted in more than 50% of the reads being aligned in both cases to the reference *L*. *mexicana* MHOM/GT/2001/U1103 genome (17,138,430 and 15,392,124 reads out of totals of 32,238,036 and 27,514,220 reads which were obtained for the WT and the AmB resistant clone, respectively, were aligned). Comparison of read coverage depth between AmB resistant and WT cells showed variations in chromosome copy numbers. An extra copy was observed to have been gained for chromosomes 05, 19, 22, and 27 and there was loss of a copy for chromosomes 12 and 17 of the AmB resistant cells as compared to the parental WT cells. In addition, a total of 5,047 single nucleotide polymorphisms (SNP) distinguish the WT and resistant lines.

Since the metabolomic data indicated changes in sterol metabolism we looked specifically for any genetic alterations in genes encoding enzymes of this pathway in AmB resistant cells. A single homozygous SNP was found among genes encoding the enzymes of the ergosterol pathway, namely in sterol 14α-demethylase (EC 1.14.13.70), where a non-synonymous mutation from A to T results in an amino acid alteration from asparagine to isoleucine (N176I) in this enzyme. Lines of several *Candida* species resistant to AmB also had mutations to sterol 14α-demethylase (ERG11) [45–47] which lead to a cessation of ergosterol production. These cells, however, were also resistant to azoles that target the demethylase whilst our *Leishmania* cell line was not. Since the *Candida* lines also accumulate lanosterol (the substrate of sterol 14α-demethylase) whilst our *Leishmania* cell line accumulated the enzyme’s product, we conclude that the *Candida* mutants have lost enzyme activity whilst our mutant retains demethylase activity but fails to provide the product into later steps of the ergosterol synthetic pathway.

**Fig 5:**
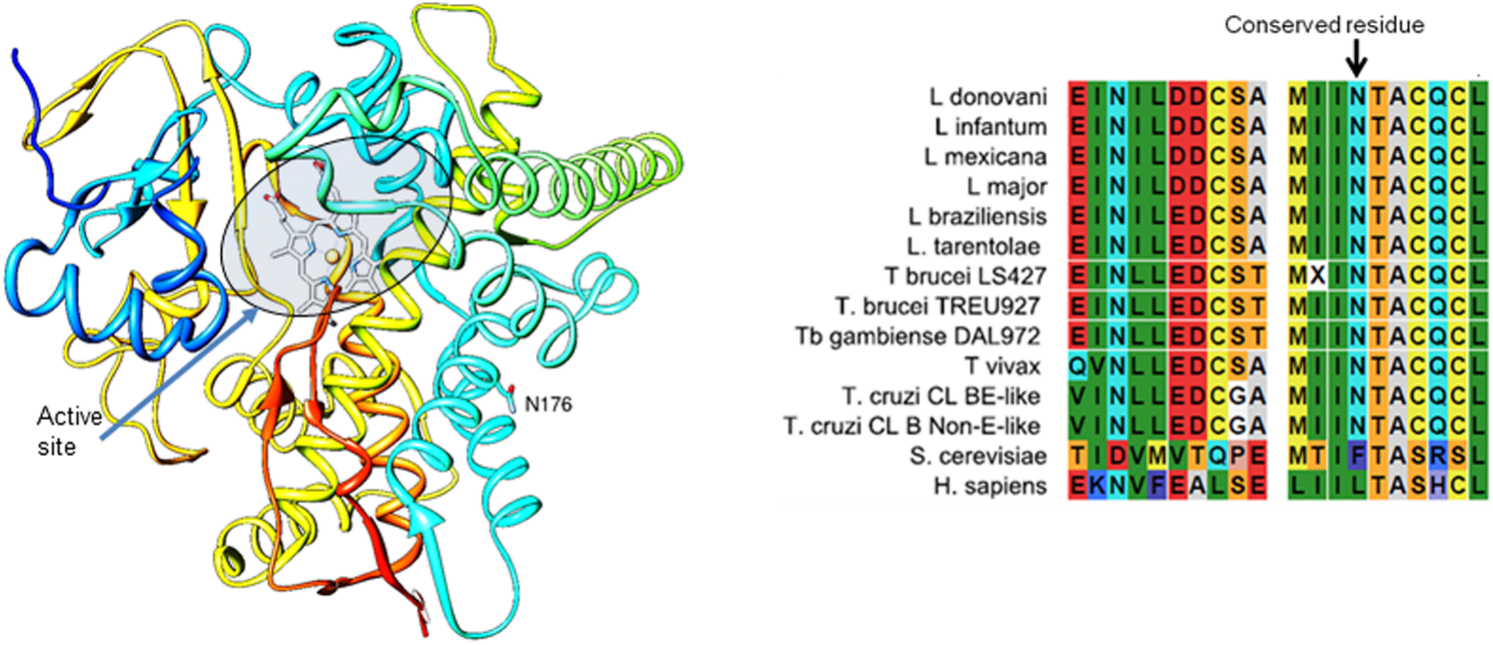
Structural and sequence context of the N176I mutation in sterol 14α-demethylase. A) A model of sterol 14α-demethylase from wild type *L*. *mexicana*. The model was built in Modeller (CCP4 Program Suite 6.3.0) based on the structure of CYP51 from *L*. *infantum*, PDB ID 3L4D, 97% amino acid sequence identity. Distal P450 view. The heme is shown as a stick model, the carbon atoms are grey, the active site area is circled. The protein ribbon is rainbow coloured from blue (N-terminus) to red (C-terminus). N176 is shown as a stick model and marked, the carbon atoms are cyan. B) Primary structure alignment of sterol 14α-demethylase of trypanosomatids, yeast, and human. The N176 residue, conserved in *Leishmania* and trypanosome species, but not in yeast and human, is indicated.

Interestingly, 4,4-dimethylcholesta-8,14,24-trien-3β-ol is the product of the sterol 14α-demethylase reaction and it was increased in the resistant cells, indicating that the enzyme itself was functional, but that its metabolic product accumulates and no longer feeds the remainder of the ergosterol pathway. Modelling of the site of the mutation revealed it to reside on an external loop of the enzyme, some way from the active site (Fig 5A). This is compatible with the enzyme’s retaining activity, but somehow becoming divorced from other features required to progress the product further through the ergosterol pathway. In *L*. *donovani*, gene knockout experiments with sterol 14α-demethylase concluded that the enzyme was essential and double knockout only possible in the presence of an expressed episomal version of the gene [48]. By contrast, null mutants were made in *L*. *major* and the cells were viable, albeit hyper sensitive to temperature stress [49].

An alignment of the primary sequences of sterol 14α-demethylase from different trypanosomatids, yeast and humans (Fig 5B) revealed that the mutated asparagine residue is conserved among trypanosomatid species analysed and not in yeast (*Saccharomyces cerevisiae*) or humans (*Homo sapiens*). The conservation could indicate important function across the Kinetoplastidae, beyond enzyme activity, for instance it could be important for protein-protein interactions.

### Expression of WT *L*. *mexicana* sterol 14α-demethylase in AmB resistant *L*. *mexicana* restores ergosterol synthesis and AmB sensitivity

**Fig 6:**
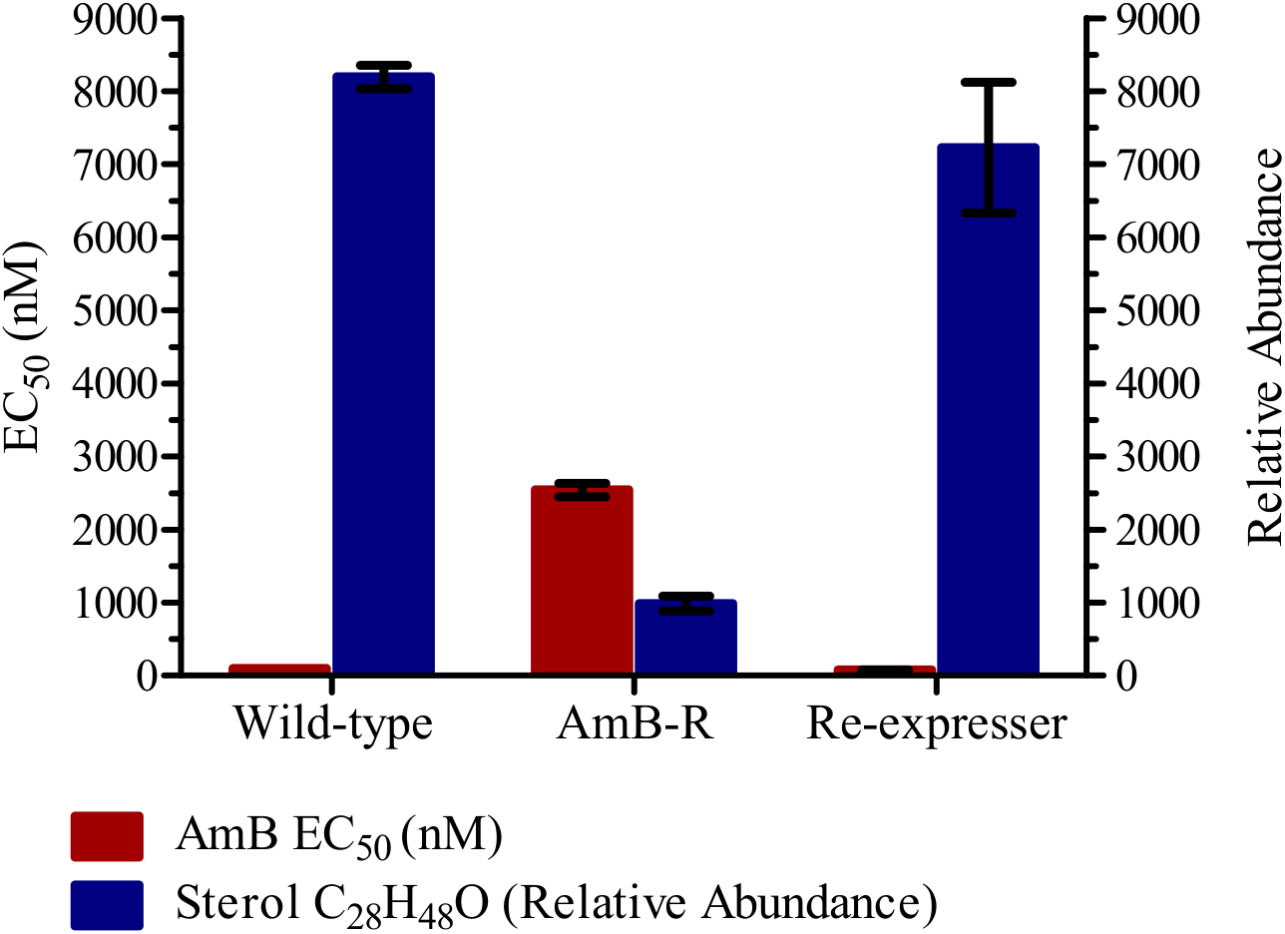
Reversal in AmB sensitivity and sterol composition after WT CYP51 re-expression in the AmB resistant cell line. Red bars represent EC_50_ values in WT, AmB resistant cells, and AmB resistant cells re-expressing WT CYP51. Mean values of three replicates are shown, error bars represent standard deviations, p < 0.0001. Blue bars represent intensity detected for a sterol C_28_H_48_O, consistent with ergosta-5,7,22E-trien-3β-ol.

Connection of ergosterol synthesis with AmB resistance was reported in previous studies, and here we observed substantial changes in the ergosterol biosynthetic pathway including loss of ergosterol, and mutation in sterol 14α-demethylase (CYP51). We therefore re-expressed the WT allele in resistant cells to see if ergosterol synthesis could be restored and whether reversion to AmB sensitivity occurred. Indeed, LC-MS revealed the restoration of the key marker of ergosterol synthesis (Fig 6), and concomitant reversion to AmB sensitivity was also associated with expression of WT CYP51 in resistant cells.

The line now expressing WT CYP51 was found to be less sensitive to ketoconazole with a fold change in EC_50_ value of 2.6 (P = 0.0072) as compared to WT cells indicating a possible over-expression of the demethylase, a known target of ketoconazole, in the re-expressor line (Table 3). The implication of overexpression would be the presence of more protein, requiring more drug to achieve the same level of inhibition of demethylase activity. The AmB resistant cells expressing the WT gene for sterol 14α-demethylase were found to have lost hypersensitivity to pentamidine by reverting back to the WT EC_50_ value for this drug and sensitivity to miltefosine was also restored.

**Table 3:** Effect of anti-leishmanial drugs on AmB resistant cells expressing the WT CYP51. Mean values of three replicates are shown with SEM values.

Drug	EC_50_ [μM]		Fold change AmB-R/WT	EC_50_ [μM]	Fold change re-expressor/WT
	WT	AmB-R		Re-expresser	
Pentamidine	4.19 ± 0.27	0.32 ± 0.01	0.08	4.18 ± 0.10	0.997
Miltefosine	5.82 ± 0.11	22.58 ± 0.20	3.88	3.591 ± 0.06	0.62
Ketoconazole	15.46 ± 0.84	24.73 ± 0.57	1.60	40.23 ± 4.82	2.60

### Subcellular localisation of sterol 14α-demethylase and sterol C14-reductase in WT and resistant lines

**Fig 7:**
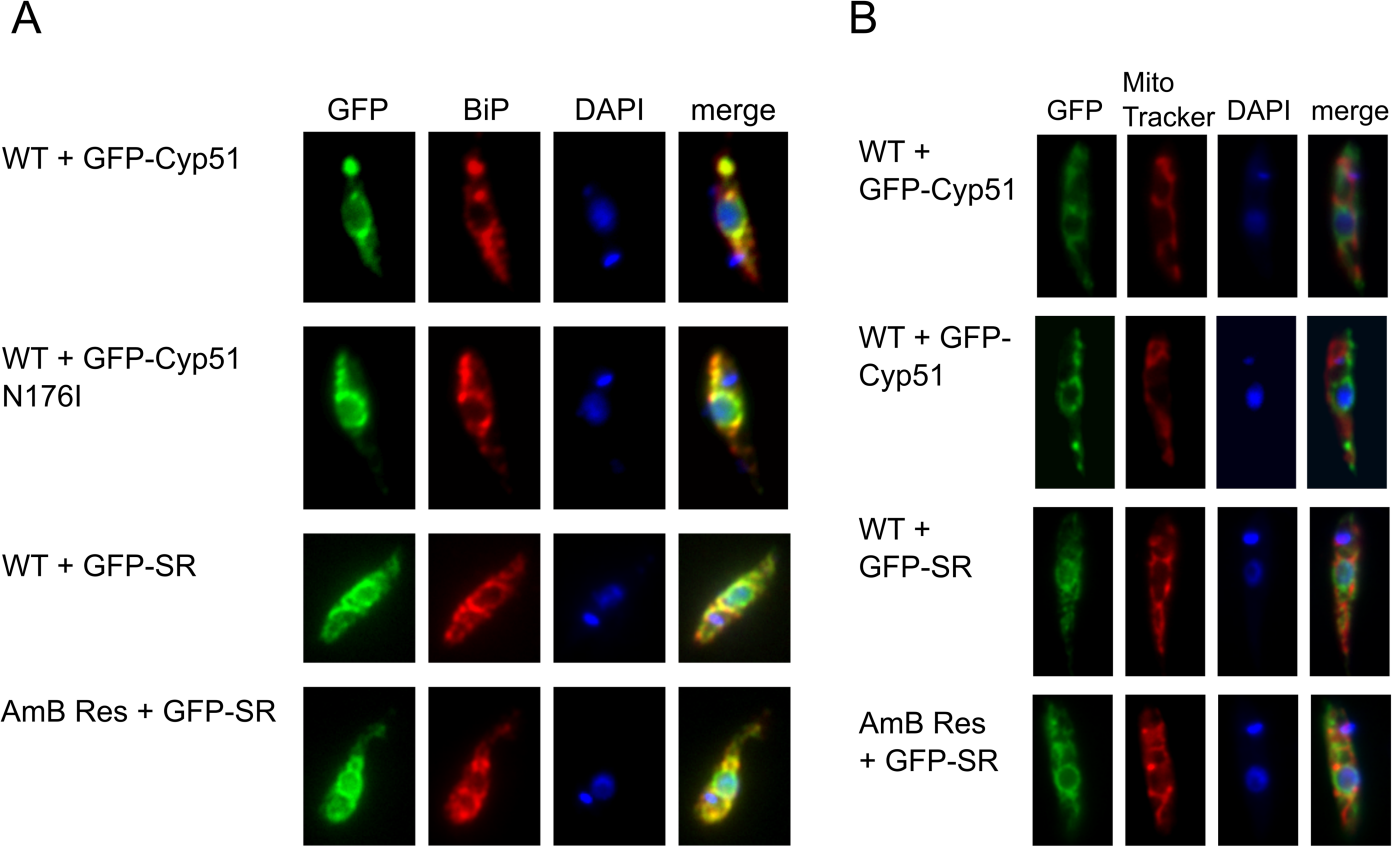
Immunofluorescence microscopy of CYP51 and sterol reductase (SR) in WT and AmBR cells. A) WT and the mutated N176I version of CYP51 were tagged with a GFP (green). α-BiP antibody was used as a marker for the endoplasmic reticulum (red; (37)). Clear overlap with the marker was detected in all cell lines tested. First line, WT expressing WT CYP51; second line, WT cells expressing N176I CYP51 (conferring resistance to AmB). SR was tagged with GFP and this construct was transfected into both WT and AmBR cells (third line, WT SR in WT cells; fourth line, WT SR in AmBR cells). Again, SR localised to the endoplasmic reticulum in both WT and AmBR cells, without any obvious difference. B) All the cell lines were probed with MitoTracker in order to test possible mitochondrial localisation. None of the tagged proteins colocalised with MitoTracker.

Since the mutation that affects sterol 14α-demethylase falls outside the active site and the enzyme retains activity as assessed by the ability of cells to convert lanosterol to its product, the mutation in the enzyme must prevent entry of the product into the remainder of the sterol pathway. Altering the subcellular localisation of the enzyme such that the product is divorced from the next enzyme in the pathway would offer a means to allow this. We therefore tested the subcellular localisation of both WT and mutant enzyme by tagging both with green fluorescent protein (GFP) at the C-terminus, the N-terminus having been proposed as important to localisation [44, 50]. A staining of the GFP-tagged CYP51 expressing cells with antibodies to the endoplasmic reticulum (ER) specific protein BiP, revealed localisation to the ER. There was no indication that the mutated enzyme localises differently from the WT enzyme at this resolution (Fig 7) hence the mutation did not seem to affect the broad compartmental localisation of the enzyme. We also tested localisation of the next enzyme in the sterol pathway, sterol C14-reductase, by tagging with GFP and it too was found in the endoplasmic reticulum in both WT and AmB resistant cells (Fig 7). The point mutation in CYP51 therefore has no effect on organellar targeting of that protein, nor the next enzyme in the pathway, although we have not been able to ascertain whether these enzymes are linked in either cell line. Higher resolution microscopy or the use of different tagging system might yield more information on the localisation of the enzymes.

## Discussion

The leishmaniases represent a significant health burden in many parts of the tropical and sub-tropical world. Elimination is a public health priority. Treatment of diagnosed patients is central to elimination plans. AmB has, in recent years, gained favour as a first line treatment for the leishmaniases, particularly in its liposomal formulation, AmBisome, which can be given in lower doses and is substantially less toxic than non-liposomal formulations of the drug. Efficacy is such that a single injection of AmBisome (10 mg/kg) is currently proposed for primary intervention [11]. A single dose regimen carries the public health benefit of assured compliance with no need for prolonged hospitalisation. However, the policy also brings with it a risk of resistance selection. Future plans for sustained therapeutic intervention with combination therapies [9, 10, 51, 52] once the best combination regimens have been chosen, might mitigate against this risk. However, where AmB is part of the combination, selection of resistance during the single shot monotherapy phase of the control programme would be calamitous.

The fact that resistance to AmB has not emerged to any great extent in the treatment of fungal infections, in spite of over 50 years of use [14, 15], coupled to various laboratory based experiments corroborating the difficulty of selecting stable AmB resistance [15] has led to the perception that the benefits of the single dose AmBisome approach outweigh the risks of resistance.

Several laboratory studies, however, have revealed that *Leishmania* can be selected for resistance to AmB, both as promastigotes and also amastigotes [23, 24]. Moreover, the first reports of parasites that are of reduced sensitivity to the drug being isolated from patients refractory to AmB are emerging [19, 20], in spite of the drug’s use against the leishmaniases having been relatively limited. Genes responsible for resistance have yet to be identified, although a number of common features have been detected in AmB resistant *Leishmania* cell lines. These include changes in sterol metabolism, where ergosterol, the primary sterol of WT *Leishmania* cell membranes is reduced or lost and replaced by different cholestane-type sterols. Enhanced ability to resist oxidative stress is also a prominent feature and proteomic analysis [19, 23, 24] has demonstrated increases in abundance of stress related proteins.

Here we set out to seek genes responsible for resistance by applying a polyomics-based approach, combining data from untargeted metabolomics analysis with whole genome sequencing. We focused on *L*. *mexicana* promastigotes which are relatively easy to work with to generate an understanding of how resistance to AmB can occur. Ultimately it will be necessary to test the relevance of these results to *L*. *donovani* amastigote forms that are responsible for visceral leishmaniasis, the primary target for AmB therapy.

We identified changes in sterol metabolism with a loss of ergosterol and could associate this with a single change to the enzyme sterol 14α-demethylase. PCR analysis of the gene encoding this enzyme from parasites selected after 50 days of drug exposure with low level resistance (Fig 1) had a WT sterol 14α-demethylase gene. By day 162, after higher level resistance had been selected, the mutant allele had appeared, indicating that lower level resistance was associated with changes other than the alteration is sterol metabolism, but the acquisition of higher level resistance required loss of ergosterol. Sterol 14α-demethylase (CYP51) has been considered an important target for chemotherapy as the azole drugs like ketoconazole and itraconazole inhibit this enzyme and show anti-leishmanial activity, albeit with disappointing results *in vivo*. Although gene knockout experiments indicated the gene was essential in *L*. *donovani* [48], recently it was shown that a *L*. *major* null mutant of sterol 14α-demethylase was viable [49]. These cells accumulated the 14-methyl sterols, 14-methyl fecosterol and 14-methyl zymosterol, and also acquired resistance to AmB, due to loss of ergosterol production. They also acquired resistance to azoles exemplified by itraconazole, which targets the demethylase. Our resistant line retained sensitivity to ketoconazole, which is explained by its having retained the demethylase activity, which also explains why the resistant line accumulates the enzyme’s product 4,4-dimethylcholesta-8,14,24-trien-3β-ol.

In yeast, the enzymes of ergosterol biosynthesis have been proposed to exist within a multi-enzyme complex, the ergosome [53]. By analogy, a similar multi-enzymatic complex could exist in *Leishmania*, although no evidence for such a complex has yet been described. To test whether the mutation we identified in sterol 14α-demethylase led to mislocalisation of the enzyme, we tagged both WT and mutant copies with GFP and followed cellular localisation. In *L*. *mexicana* the enzyme is found primarily in the ER as in *L*. *major* [49].This localisation is retained in both WT and resistant lines. No gross change in localisation of the following enzyme, sterol C14-reductase, is apparent when mutated CYP51 N176I is expressed. It seems likely, therefore, that the mutation, instead, prevents an interaction with this or another protein and this alteration may prevent the channelling of the product into the subsequent reactions of ergosterol synthesis leading to accumulation of 4,4-dimethylcholesta-8,14,24-trien-3β-ol and other intermediates. The accumulated sterols are presumably sufficient for key roles of sterols in the resistant cell lines. *Leishmania* therefore may contain a multi-enzyme ergosome analogous to that described in yeast [53] and direct protein interactions may be essential for the proper function. We plan to investigate the presence and composition of the leishmanial ergosome in future work

It was also of note that the AmB resistant line we selected was hypersensitive to oxidative stress (created by hydrogen peroxide and methylene blue) and to pentamidine, an anti-leishmanial drug previously indicated to exert its mode of action through induction of oxidative stress [54, 55]. Hypersensitivity to hydrogen peroxide, methylene blue and pentamidine was reversed along with the AmB resistance phenotype upon expression of the WT demethylase gene. Possible explanations for this increase in sensitivity to oxidative stress include changes to the cell membrane integrity and fluidity after loss of ergosterol, as was observed previously [19, 24], or possibly the enhanced capability of ergosterol itself as an agent of protection against oxidative stress [56]. Increases in stress-response proteins have also been reported in other AmB resistant lines [22, 25]. It is possible that this relates to their selection leading to loss of ergosterol and the concomitant increase in sensitivity to oxidative stress is secondarily compensated by additional changes to enzyme pathways dealing with oxidative stress. It is important to note here that other leishmanicides may lead to selection of enhanced resistance to oxidative stress [57], hence these stress tolerant parasites might form resistance to AmB relatively readily. Even environmental pressures, such as high levels of arsenic in drinking water, can lead to selection of antimony resistance and reduced oxidative stress sensitivity [58]. The potential of *Leishmania* strains, pre-adapted in ways that will allow relatively easy selection for AmB resistance is therefore of significant concern.

Resistance to AmB in this study therefore relates to changes in the sterol composition of the parasite’s membrane with AmB binding ergostane-type sterols replaced by less avidly binding cholestane-type sterols. This is achieved, in this instance, by mutating an enzyme, sterol 14α-demethylase, of the sterol synthesis pathway in a manner which affects not its active site but its ability to channel its enzymatic product to subsequent steps of the pathway. A survey of the literature describing other AmB resistant *Leishmania* indicates that loss of ergostane sterols is a common step in development of resistance to the drug [19, 23, 24]. However, it appears likely that different mutations to various enzymes in the pathway can contribute. The fact that treatment failures with AmB are reported in India and in at least one case sterol metabolism is changed [19] points to a necessity to contemplate spread of resistance to this drug. The frequency with which changes to sterol composition emerge does point to possible tests to survey for resistance. In addition to seeking different genes whose mutation can cause resistance, simple tests for sterol composition including spectrophotometric discrimination between ergosterol and cholestane-type sterols [59, 60] offer approaches with which to develop tests for resistance to the drug. Mutations in sterol 14α-demethylase can also be identified, but other mutations too might also provide the same ultimate result of loss of ergosterol synthesis and further analysis of genes associated with resistance lines will enhance understanding in this area.

## Supporting information

S1 Table IDEOM file for metabolomic comparison of *L*. *mexicana* wild type and derived amphotericin B resistant cell line. (XLSX)

S1 Fig Modified Alamar Blue replicate comparison plots for the response to amphotericin B (AmB) and pentamidine by *L*. *mexicana* promastigote wild-type (Wt) and derived Amphotericin B resistant cells (AmBR). The graphs in the first column are three replicates for AmB comparison and the second column is for three replicates for pentamidine. Graphs were plotted using GraphPad Prism 5.
(PDF)

S2 Fig Modified Alamar Blue replicate comparison plots for the response to ketoconazole and miltefosine by *L*. *mexicana* promastigote wild-type (Wt) and derived Amphotericin B resistant cells (AmBR). The graphs in the first column are three replicates for response to ketoconazole and the second column is for three replicates for miltefosine. Graphs were plotted using GraphPad Prism 5.
(PDF)

S3 Fig Modified Alamar Blue replicate comparison plots for the response to potassium tartrate antimony (PAT) representing SbIII by *L*. *mexicana* promastigote wild-type (Wt) and derived Amphotericin B resistant cells (AmBR) in the first column. The second column shows comparison of the response to pentamidine by AmBR and re-expressor cells, indicating the reversion to wild-type tolerance of the drug. Note that comparison of the tolerance to pentamidine by Wt and AmBR is shown on S1 Fig. Graphs were plotted using GraphPad Prism 5.
(PDF)

S4 Fig Modified Alamar Blue replicate comparison plots for the response to miltefosine by AmBR and Re-expressor cells in the first column–comparison for the Wt and AmBR is shown on S2 Fig. The second column shows comparison of the response to ketoconazole by *L*. *mexicana* promastigote Wt, derived AmBR and re-expressor cells. Graphs were plotted using GraphPad Prism 5.
(PDF)
