# Supplementary figures and images for: Sterol 14α-demethylase mutation leads to amphotericin B resistance in Leishmania mexicana
Source: PLoS Negl Trop Dis. 2017 Jun 16;11(6):e0005649. doi: 10.1371/journal.pntd.0005649 (PMC5498063; doi:10.1371/journal.pntd.0005649)

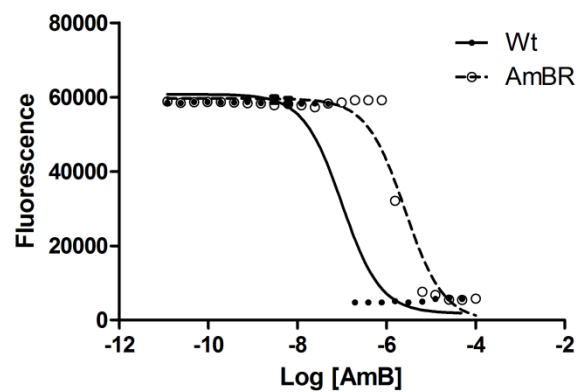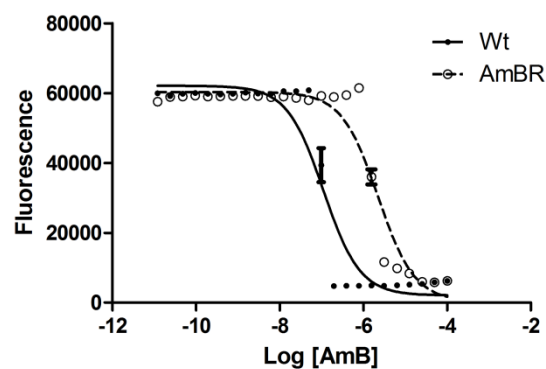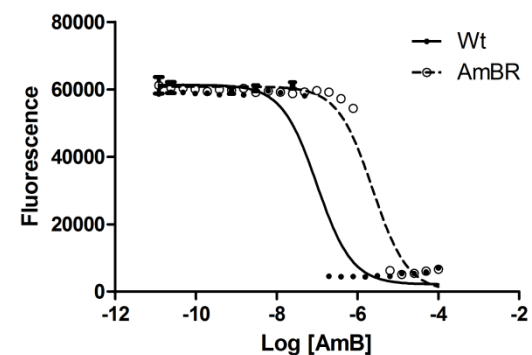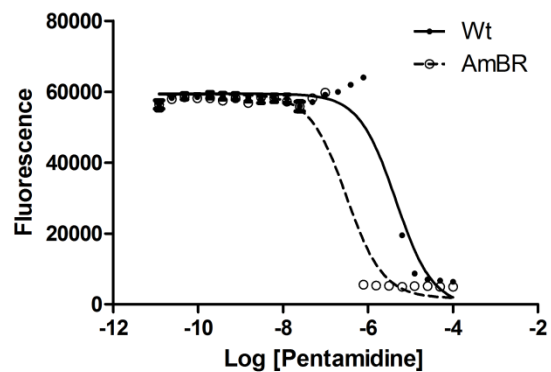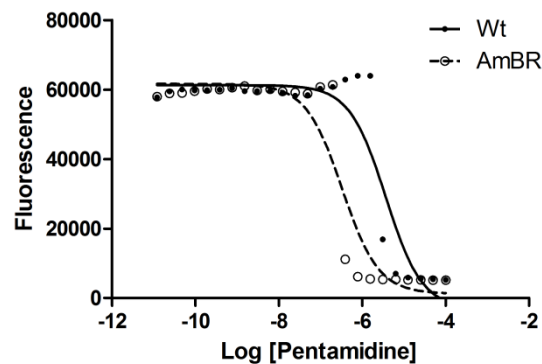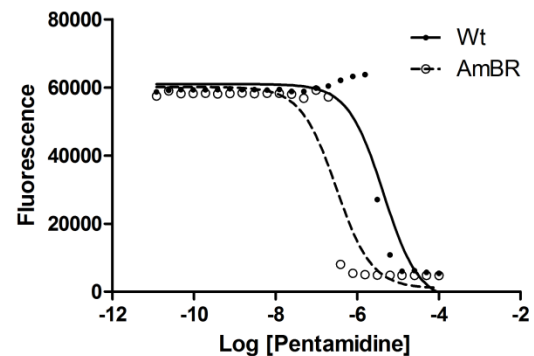

Supplement: S1 Fig — The graphs in the first column are three replicates for AmB comparison and the second column is for three replicates for pentamidine. Graphs were plotted using GraphPad Prism 5. (PDF) [file pntd.0005649.s002.pdf]

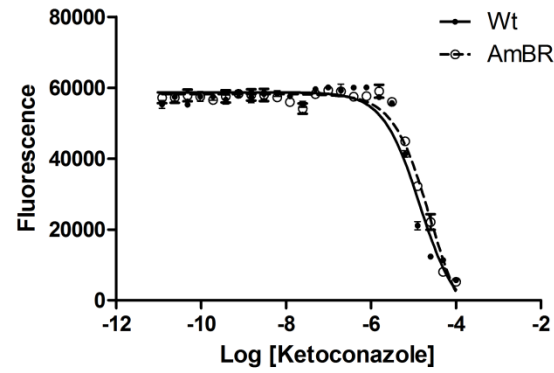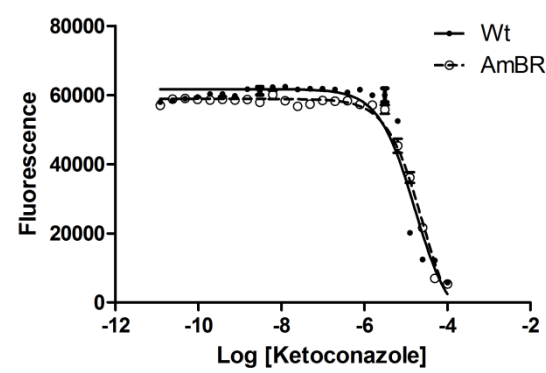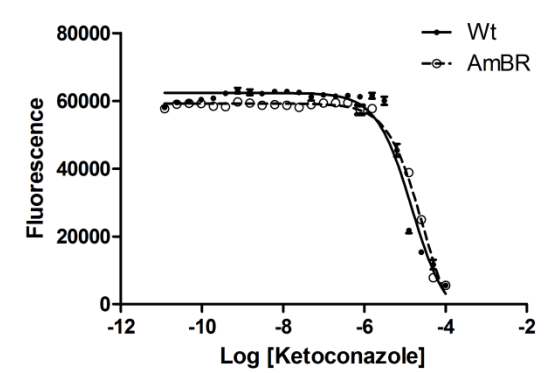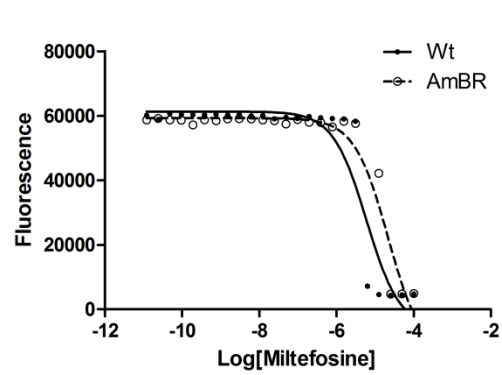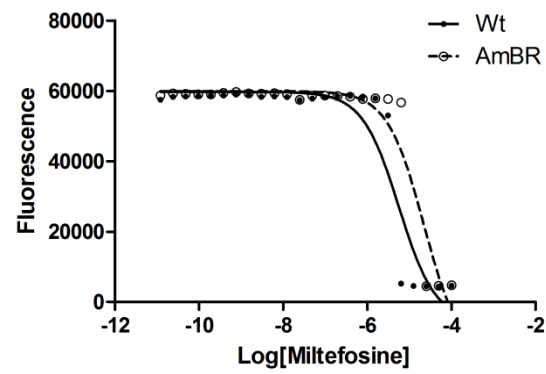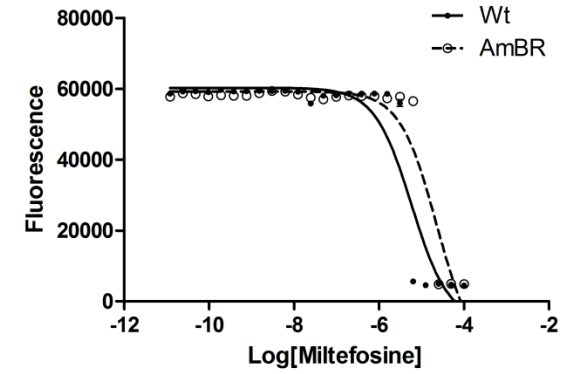

Supplement: S2 Fig — The graphs in the first column are three replicates for response to ketoconazole and the second column is for three replicates for miltefosine. Graphs were plotted using GraphPad Prism 5. (PDF) [file pntd.0005649.s003.pdf]

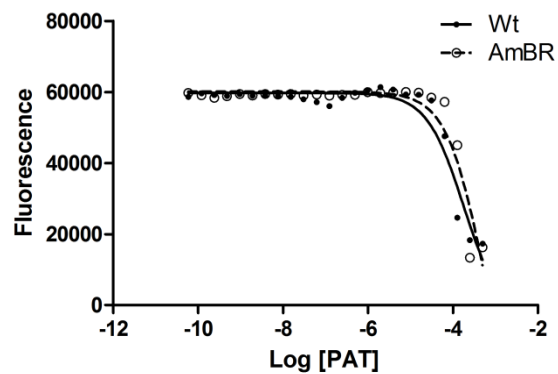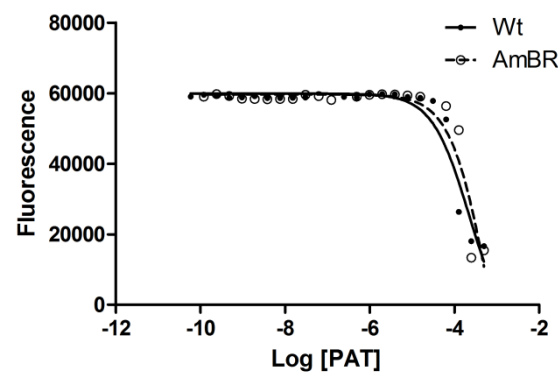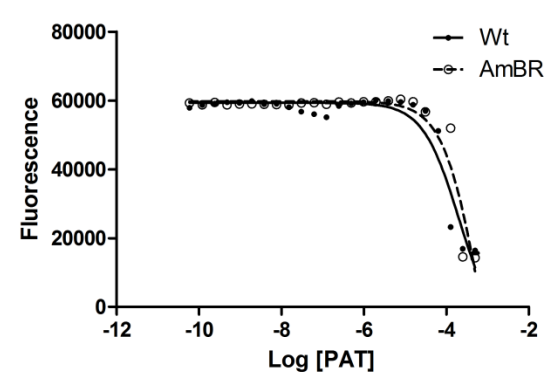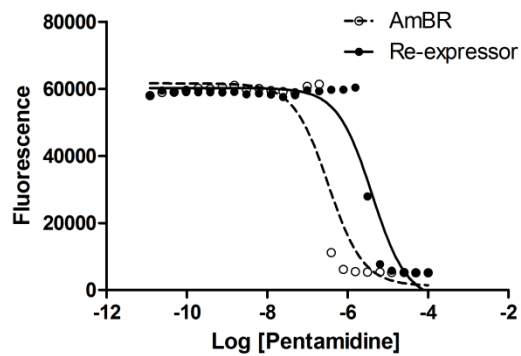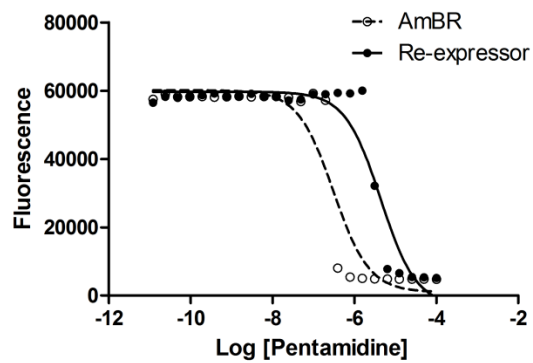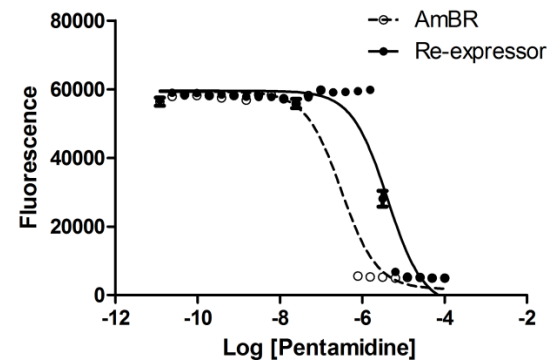

Supplement: S3 Fig — The second column shows comparison of the response to pentamidine by AmBR and re-expressor cells, indicating the reversion to wild-type tolerance of the drug. Note that comparison of the tolerance to pentamidine by Wt and AmBR is shown on S1 Fig. Graphs were plotted using GraphPad Prism 5. (PDF) [file pntd.0005649.s004.pdf]

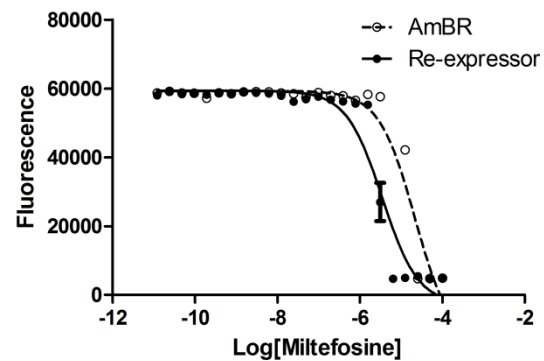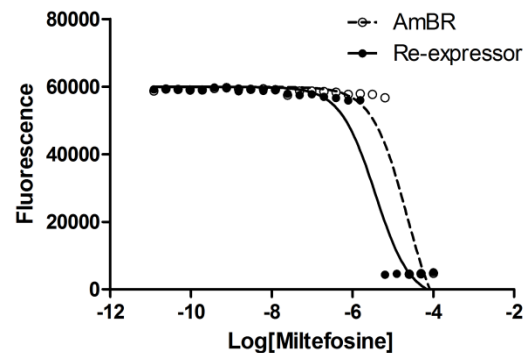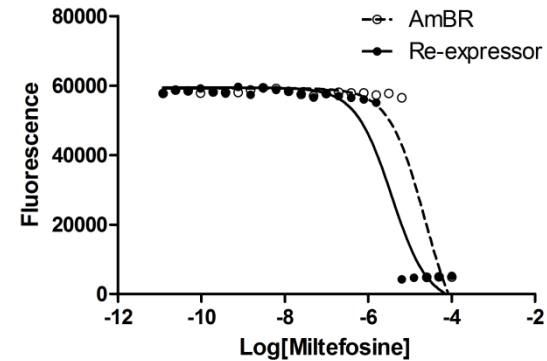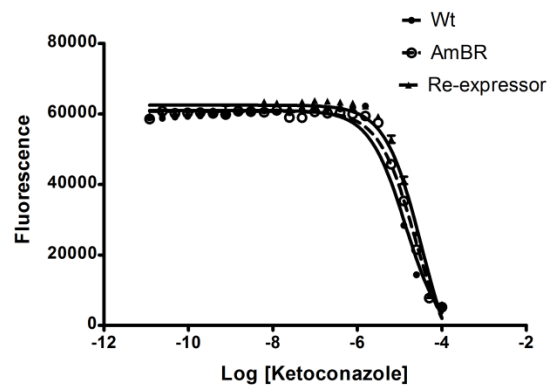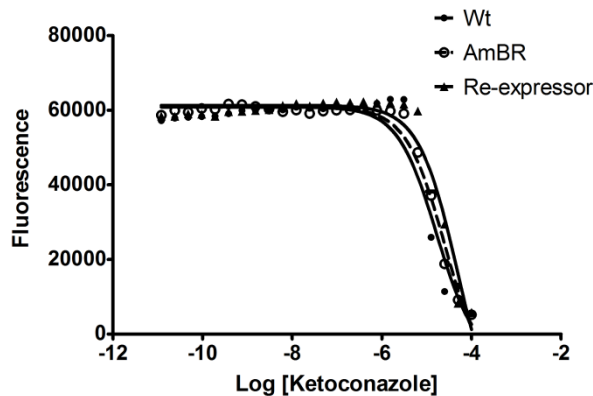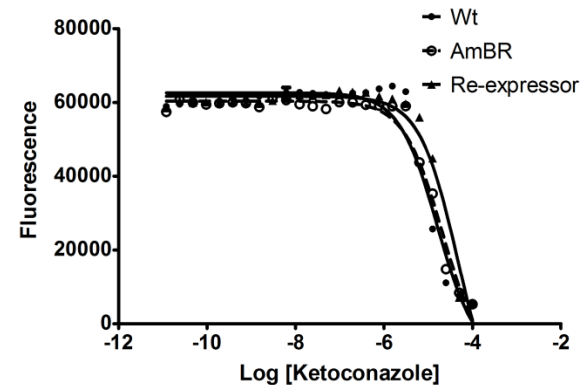

Supplement: S4 Fig — The second column shows comparison of the response to ketoconazole by L. mexicana promastigote Wt, derived AmBR and re-expressor cells. Graphs were plotted using GraphPad Prism 5. (PDF) [file pntd.0005649.s005.pdf]
